# Proceedings of the American Pharmaceutical Association

**Published:** 1861-03

**Authors:** 


ART. VIII.—*Proceedings of the American Pharmaceutical Association.* 8vo., pp. 278. Philadelphia, 1860.

THIS volume, composed of the transactions of the American Pharmaceutical Association at the ninth annual meeting, held in the City of New York, September, 1860, contains, besides the address of the retiring president, a large number of reports, which are replete with interest and valuable information to the pharmaceutist, as well as to the practitioner of medicine. Among the volunteer papers, we have read with pleasure the very interesting memoir of the Hon. Henry Cavendish, by Henry F. Fisk, and regret that our limited space does not admit of our transcribing some of the leading points in the character and writings of that eccentric philosopher.

